# Effects of Pregnancy Loss on Subsequent Postpartum Mental Health: A Prospective Longitudinal Cohort Study

**DOI:** 10.3390/ijerph18042179

**Published:** 2021-02-23

**Authors:** David C. Reardon, Christopher Craver

**Affiliations:** 1 Elliot Institute, St. Peters, MO 63376, USA; 2 Charlotte Lozier Institute, Arlington, VA 22206, USA; ccraver@lozierinstitute.org

**Keywords:** perinatal mental health, postpartum psychiatric treatments, pregnancy loss, abortion, miscarriage

## Abstract

Pregnancy loss, natural or induced, is linked to higher rates of mental health problems, but little is known about its effects during the postpartum period. This study identifies the percentages of women receiving at least one postpartum psychiatric treatment (PPT), defined as any psychiatric treatment (ICD-9 290-316) within six months of their first live birth, relative to their history of pregnancy loss, history of prior mental health treatments, age, and race. The population consists of young women eligible for Medicaid in states that covered all reproductive services between 1999–2012. Of 1,939,078 Medicaid beneficiaries with a first live birth, 207,654 (10.7%) experienced at least one PPT, and 216,828 (11.2%) had at least one prior pregnancy loss. A history of prior mental health treatments (MHTs) was the strongest predictor of PPT, but a history of pregnancy loss is also another important risk factor. Overall, women with a prior pregnancy loss were 35% more likely to require a PPT. When the interactions of prior mental health and prior pregnancy loss are examined in greater detail, important effects of these combinations were revealed. About 58% of those whose first MHT was after a pregnancy loss required PPT. In addition, over 99% of women with a history of MHT one year prior to their first pregnancy loss required PPT after their first live births. These findings reveal that pregnancy loss (natural or induced) is a risk factor for PPT, and that the timing of events and the time span for considering prior mental health in research on pregnancy loss can significantly change observed effects. Clinicians should screen for a convergence of a history of MHT and prior pregnancy loss when evaluating pregnant women, in order to make appropriate referrals for counseling.

## 1. Introduction

Pregnancy loss (natural or induced) is associated with an increased risk of mental health problems [1,2,3,4]. Self-reports and clinical experience suggest that unresolved and suppressed feelings regarding prior pregnancy losses may be aggravated or triggered by subsequent pregnancies [5,6]. At least some women report that their feelings of joy regarding a pregnancy they are carrying to term are comingled with a feelings of loss, impacted grief, and or guilt regarding prior pregnancy losses [5]. Others report heightened fears about losing the subsequent pregnancy and/or fears of being unworthy to be a mother, and other anxieties which sometimes impede bonding [5]. These anecdotal observations of a link between pregnancy loss and subsequent mental health problems during the antenatal and postpartum periods of subsequent pregnancies are supported by statistical evidence from a small number of studies. These studies have revealed that pregnancy loss is associated with higher rates of mental health treatments during both the antenatal [7,8,9,10] and the postpartum period [11,12,13,14,15,16]. Moreover, at least one study has revealed that fear of childbirth is a significant risk factor for postpartum depression [14], which is consistent with reports of heightened fears surrounding subsequent wanted pregnancies among women with a history of induced abortions [5]. Most studies examining the effects of pregnancy loss on maternal mental health, however, are limited to relatively small sample sizes and often lack a comprehensive control for prior mental health issues. Therefore, the primary purpose of this study is to examine both the effects of pregnancy loss and prior mental health on treatment rates for postpartum mental illness in a large population of primiparous women. Since early intervention can ameliorate the symptoms of perinatal depression [17], screening for a history of pregnancy loss may lead to earlier referrals to address unresolved mental health issues.

A secondary purpose is to examine the differences associated with the time frames used for considering the effects of prior mental on mental health.To date, there does not appear to be an evidence-based standard for determining which, if any, time frames are best suited for understanding the effects of prior mental health on mental health during and after a subsequent pregnancy. The choice of time frames used to consider prior mental health appears to vary widely, jumping about from nine months [18] to one year [19] or two years [20], even among research teams employing the same individuals. Therefore, our secondary purpose in this paper is to investigate different time frames before, between, and during pregnancy, in order to better understand how the choice of time frame may affect findings and the interpretation of results relative to reproductive mental health issues.

## 2. Materials and Methods

### 2.1. Study Population

Data was obtained from the United States Centers for Medicare and Medicaid Services (CMS) using the data submitted to CMS from the 17 states (Alaska, Arizona, California, Connecticut, Hawaii, Illinois, Maryland, Massachusetts, Minnesota, Montana, New Jersey, New Mexico, New York, Oregon, Vermont, Washington, and West Virginia) where Medicaid coverage included all reproductive health care options, including induced abortion, during the years 1999 through at least 2012, inclusive. Data for each beneficiary was rolled in beginning in 1999 or the year of each woman’s 14th birthday, whichever was later. The study population was limited to all women born in 1983 or later who had at least one live birth over the age of 13 between 1999 and June of 2012 inclusive, and had been eligible for Medicaid coverage for at least 12 months between 1999 and 2012. Using these selection criteria, wherein the oldest women in the cohort were 16 years of age in 1999 and 29 years of age in 2012, maximized the likelihood that our data captured the first pregnancy outcome for the vast majority of our study population.

### 2.2. Study Variables

The primary outcome variable was any postpartum psychiatric treatment (PPT), defined as any treatment code associated with the International Classification of Diseases (ICD-9) codes 290–316 occurring within six months of each woman’s first known live birth. In addition, beneficiaries who had at least one inpatient PPT or one emergency room PPT were identified for a subgroup analysis.

In addition, for each woman, her race, age at first live birth, and the date and outcomes of all pregnancies prior to and including her first live birth, were also extracted. To address the secondary analysis examining the effects of time frames used to control for prior mental health, any occurrence of a mental health treatment (MHT), defined as any ICD-9 codes 290–316, was identified for each of the following periods: one year before the first conception date, one year prior to the first pregnancy outcome, one year prior to the first live birth, any time prior to the first conception, any time prior to the first pregnancy outcome, any time prior to the first live birth, between first conception and the first live birth, and any time after the first conception but prior to the first live birth.

### 2.3. Identification of Pregnancies and Conception Dates

Pregnancy outcomes were identified using diagnostic ICD-9 codes and clarified with Current Procedural Terminology (CPT) codes and Healthcare Common Procedure Coding System (HCPS codes. Multiple diagnostic or treatment codes for any pregnancy within 30 days of other pregnancy codes were collapsed into a single pregnancy outcome, using the first date associated with that cluster of Medicaid claims. Pregnancy outcomes were segregated into four categories: live birth; induced abortion; natural fetal loss (miscarriage, ectopic pregnancy, molar pregnancy, or stillbirth); and indeterminate loss, wherein the latter combined missed abortion (ICD 632; *n* = 28,859), unspecified abortion (ICD 637; *n* = 12,655), illegally induced abortion (ICD 636; *n* = 271), and failed attempted abortion (ICD 638; *n* = 0). All women with any history of an induced abortion, natural loss, or indeterminate loss prior to their first live birth were identified as having a pregnancy loss. Also, to address coding errors or other conflicts with the data, coding indicating an abortion within 36 weeks prior to a live birth was excluded, as well as any data indicating an abortion or natural loss within four weeks of an induced abortion. The estimated date of conception was calculated for each pregnancy by subtracting 290 days from the date of a livebirth and 84 days from the date of a pregnancy loss.

### 2.4. Statistical Analyses

Logistic regression analyses were conducted to compare the subsets of women who experienced PPT to those women who did not. Covariates included age, race, type and number of losses, and history of MHTs. Regarding the history of MHTs, several models were run to examine any differences between each of the following time frames: one year prior to first conception; any time prior to first conception; one year prior to first pregnancy outcome; any time prior to first pregnancy outcome; one year prior to first live birth; any time prior to first live birth; and for the interval between the first conception and first live birth.

## 3. Results

Using the selection criteria for young Medicaid beneficiaries described above, we identified the first live birth of 1,939,078 Medicaid beneficiaries. Of these women, 207,654 (10.7%) experienced at least one postpartum psychiatric treatment (PPT) within 6 months of the delivery, and 216,828 (11.2%) had at least one pregnancy loss prior to their first live birth. Overall, younger women experienced higher rates of PPT, 12.9%, 9.7%, and 8.6% for women aged 14–19, 20–24, and 25–29, respectively, but the risk of PPT following an earlier pregnancy loss increased with age. There were even more significant variations relative to race. Overall, Hispanic women had the lowest rate of PPT, at 5.5% compared to 15.9%, 9.3%, and 7.8% for whites, Blacks, and other races, respectively.

Table 1 shows the rates of PPT in various subgroups segregated by exposure to a pregnancy loss prior to the first birth. Overall, the 216,828 women with one or more pregnancy losses prior to their first live births were about 35% more likely to require PPTs than women who delivered their first pregnancies. Relative to race, the risk of PPT associated with pregnancy loss increased 19% for Black women, 32% for white women, 59% for Hispanic women, and 48% for other races. Overall, PPT treatment was twice as likely to occur within the first 90 days after delivery compared to the next 90 days, but a history of pregnancy loss was associated with a 42% increased risk of PPT within the first three months and a 21% increased risk in the second three months. In addition, while most women experiencing PPT received outpatient treatment only, the risk of inpatient treatment was 83% higher for those with a history of pregnancy loss. There was also a 22% higher risk that PPT treatment was sought at emergency room.

**Figure 1:**
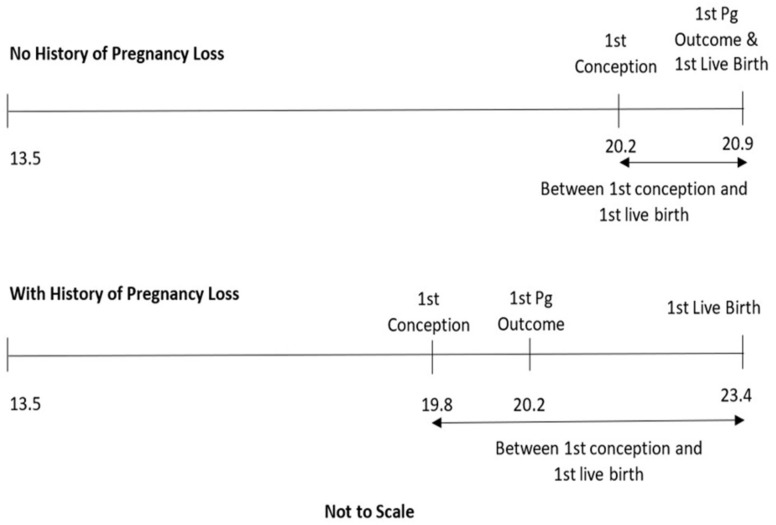
Mean average age of women at entry into cohort, first conception, first pregnancy (Pg) outcome, and first live birth, segregated by any history of pregnancy loss. Not to scale.

Table 2 shows the treatment rates between the two groups relative to their history of receiving mental health treatments (MHTs) prior to their first live birth. To address our secondary research objective, multiple time frames were examined. The time frames of interest are illustrated in Figure 1. The mean average age of at first conception was 19.8 (standard deviation: SD = 3.0) for those whose first pregnancy was a loss, and 20.2 (SD = 2.9) for those whose first pregnancy was delivered. The average age of first live birth was 23.4 (SD = 3.8) for those with a history of one or more losses, and 20.9 (SD = 2.8) for those without a history of loss. The average age of women as they were rolled into the data cohort was approximately 13.5 years of age. Therefore, the time frame for “any time” prior to a specific date in Table 2, Table 3 and Table 4 includes the time between 13.5 years of ag and the specified reference date.

Table 2 reveals that the choice of time frame (see Figure 1) can significantly change the strength of associations between pregnancy loss and the outcome variable. For example, among women with at least one MHT during the year prior to their first live birth, nearly half required PPT regardless of pregnancy loss history. However, when the period of observation of prior MHT was one year prior to their first pregnancy loss, nearly 100% of women required PPT following their first live birth.

Among women with no history of mental health problems in the selected time frames, pregnancy loss was positively associated with PPT (OR > 1) in all but one case. The one exception was in the comparison of women with no history of MHT any time prior to their first live birth. With this subgroup, women with and without a history of pregnancy loss had nearly identical rates of PPT (7.03–7.05%). However, for those with any history of MHT, the risk of PPT was four times higher (27.69–30.73%). Notably, with the exception of two cases, among women with a history of MHT pregnancy loss was inversely associated with PPT (odds ratio:OR < 1). These findings may indicate either that women with a pregnancy loss are more likely to have a successful history of mental health care, which helps to reduce the need for PPT, or these findings may reflect an emotional boost or healing effect associated with experiencing a successful birth following a pregnancy loss. Of the two exceptions, only one is notable, but it is very notable: MHT one year prior to a first pregnancy ending in a loss was almost perfectly correlated with PPT (99.97%).

Table 3 shows the results of two regression analyses examining the risk of PPT in the entire population studied. Model 1 examines the effects of a history of pregnancy loss and demographic characteristics. This analysis revealed that the experience of a prior pregnancy loss increased risk of PPT by 27% (adjusted OR = 1.27; 95% CI = 1.25–1.29), non-white women had half, or less, of the PPT rates compared to white women, and younger women were at greater risk of PPT. Model 2 includes additional variables relative to the women’s prior history of mental health treatments. Notably, Model 2 shows that a history of MHT between a first conception (which may end in a pregnancy loss) and a first live birth has the strongest effect (adjusted OR = 13.39; CI = 13.16–13.62). This strong effect is at least in part explained by the fact that women with a pregnancy loss had a much longer period of time in which MHT may have been provided than women who carried their first pregnancies to term.

Table 4 shows the results of two additional regression models examining the subset of women with a history of pregnancy loss. Both models examine the effects of demographics, the number of pregnancy losses, the interval between the first pregnancy loss and the first live birth, and the type of the first pregnancy loss on PPT rates. Model 3 also examines the effects relative to one-year periods prior to the first live birth, first conception, first pregnancy outcome, and the time period between the first conception and first live birth. In order to examine a longer time span of prior mental health, alternatively, Model 4 controls for any time prior to first live birth, any time prior to first conception, any time prior to the first pregnancy outcome, and the time period between the first conception and first live birth. Both models reveal that the number of losses and type of loss had little effect. The shorter interval between the first pregnancy loss and first live birth (<24 months) had a small but significant effect in Model 3, but this significance was not present by Model 4. The time periods for examining MHT that had the highest adjusted odds ratios were one year prior to the first live birth (1.75), one year prior to the first pregnancy outcome (>999.999), the interval between first conception and first live birth (3.9 in Model 3 and 7.25 in Model 4), and any time prior to the first conception (2.29).

## 4. Discussion

Our findings reveal that prior pregnancy loss and a history of prior mental health treatments (MHT), individually and especially in combination, are risk factors for postpartum psychiatric treatments (PPT). Overall, pregnancy loss prior to a first live birth increases the risk of postpartum psychiatric disorders, both before and after controlling for prior mental health history. This risk is most elevated in the first 90 days postpartum (OR = 1.42) and for inpatient treatments (OR = 1.83). These findings are consistent with the only other population study we could identify that examined both pregnancy loss and prior mental health as risk factors for postpartum depression [14].

Among women with no history of MHT, pregnancy loss is consistently associated with elevated risk of PPT (Table 2). Still, the strongest observed predictor of PPT is a history of MHT. Yet, for women with a history of MHT, the relationship with PPT is complicated by any exposure to pregnancy loss and specific time frames in which prior MHT are considered.

Our use of multiple models demonstrates that both the recency of MHT (Model 3) and any history of MHT (Model 4) show significant effects that would have been missed if only a single model was employed. For example, MHT one year prior to the first pregnancy outcome (including losses) was almost perfectly correlated to PPT following a first live birth, but MHT any time prior to the first pregnancy outcome was only weakly correlated (Adj OR = 1.09). Conversely, any time prior to first conception was nearly twice as predictive as one year prior to first conception.

Another notable finding revealed by our use of multiple time frames was that the risk of PPT is modestly reduced among women with a history of both MHT and pregnancy loss (see Table 2). This may be due to either a successful history of mental health treatments that prepare some women to handle postpartum stress. or to positive effects associated with giving birth following a pregnancy loss. A very notable exception was found in cases where mental health treatments were provided within one year prior to the pregnancy loss, in which case nearly 100 percent of the women required PPT. When examining only women with PPT, Model 4 (Table 4) shows that the strongest effect was correlated to a history of MHT in the interval between conception of a first pregnancy and a first live birth (Adj OR = 7.25), which includes not only the nine months of a pregnancy (indicating the importance of recency) but also the period of time following any pregnancy losses preceding the birth. Notably, this time between first conception and first birth was the second most powerful predictor in Model 3.

These findings are especially important in regard the interpretation of existing literature on postpartum psychiatric disorders. In general, insufficient attention has been given to the impact of pregnancy loss on subsequent postpartum psychiatric events [16,17]. Furthermore, as seen in Table 2, Table 3 and Table 4, the choice of time frame used for consideration of prior MHT can profoundly change results. Meaningful effects may be missed, or obscured, in studies [18,19,20] that fail to distinguish between MHT events both before and after prior pregnancy losses. Therefore, research into the interactions between reproductive and mental health should include the consideration of multiple time frames regarding prior history of mental health treatments. These time frames account for recency of prior mental health issues, lifetime exposure to mental health treatments, and the history of any mental health treatments following pregnancy loss(es).

A number of limitations apply to this study. First, a history of seeking mental health care is likely an indicator of a greater willingness to seek postpartum mental health care. Conversely, many women who may benefit from MHT may simply not seek it either before or after their first live birth. Similarly, the differences observed in relation to race may be artifacts of different levels of cultural acceptance of MHT. Second, the available data was limited to low-income women. In part, this is an advantage, since it eliminates the likelihood that the differences observed are due to socioeconomic factors. Still, additional research is necessary to confirm that the differences observed exist across all income classes. Third, Medicaid eligibility changes with age, circumstance, across states, and across different fiscal years, which can create data gaps. The effects of such churning, however, were reduced by excluding women with less than 12 months of eligibility. Moreover, since pregnancy increases eligibility for Medicaid, it is likely that most pregnancies subject to any medical treatment were identified in the medical records for women in this economic group. Fourth, early miscarriages can often occur without any medical treatment. However, unless early miscarriages are linked with a significantly decreased risk of PPT, which is unlikely, more complete data on miscarriages would likely strengthen rather than weaken the findings regarding elevated risk of PPT when there is a history of pregnancy loss. Fifth, this study only examines PPT risks following a first live birth. While it seems likely that similar effects would be observed following subsequent live births, additional research is necessary to address the effects relative to the number of live births.

Given the limitations on our data, it would be beneficial in future research to examine the effects of relationship status, education, employment, pregnancy intention, and risk factors relevant to induced abortion, such as coercion or emotional attachment to the pregnancy [18]. Additional research should also be conducted to investigate specific diagnoses associated with PPT relative to prior pregnancy loss. Another research objective should be the evaluation of interventions and counseling programs for pregnancy loss that may ameliorate the increased risk of PPT following a live birth.

## 5. Conclusions

A history of pregnancy loss is an independent risk factor for postpartum psychiatric illness. This risk is heightened by a co-occurring history of mental health treatments. Important differences are observed in relation to the timing of mental health treatments occurring before and after a pregnancy loss and the relative risk of subsequent PPT. 

Important clinical implications arise from this study. Both a history of mental health treatments and prior pregnancy loss are risk factors for PPT, especially when both are present. Clinicians should be alert to these findings, in order to better identify and refer women at higher risk to appropriate counseling. Moreover, for mental health counselors, the appearance of postpartum disorders may present an opportunity to help patients address underlying issues. The clinical experience of grief counselors has revealed that many women will not offer to discuss prior pregnancy losses unless invited to do so [19]. A simple, “Do you have any unresolved feelings about any prior pregnancy losses that you would like to discuss?” may serve as the invitation some women may need to open up about sensitive or difficult topics.

## Figures and Tables

**Table 1:** Total of all women by subgroup with percentages of total receiving postpartum psychiatric treatment (PPT) rates relative to first pregnancy (Pg) outcome, population differences, timing, and type of treatment.

Characteristics of Study Population	No Prior Pg Loss		Yes Prior Pg Loss		Crude Odds Ratio
	*n* = 1,722,250		*n* = 216,828		
	% Receiving PPT	Total	% Receiving PPT	Total	
Total	10.36%	1,722,250	13.50%	216,828	1.35
Age at live birth (years)					
14–19	12.65%	603,925	14.24%	96,119	1.15
20–24	9.34%	885,674	13.13%	97,343	1.47
25–29	8.29%	232,651	12.03%	23,366	1.51
Calendar year of live birth					
2000–2002	9.98%	638,645	13.69%	36,350	1.43
2003–2005	10.50%	448,784	13.69%	51,142	1.35
2006–2008	10.64%	472,841	13.26%	58,188	1.28
2009–2011	10.62%	161,980	13.47%	71,148	1.31
Race					
White	15.43%	696,577	19.38%	84,731	1.32
Black	9.08%	315,128	10.60%	62,161	1.19
Hispanic	5.22%	485,319	8.05%	46,334	1.59
Other	7.52%	225,226	10.74%	23,602	1.48
First occurrence of PPT					
Within 90 Days	7.30%	1,665,464	10.05%	208,498	1.42
Within 91–183 Days	3.55%	1,600,662	4.25%	195,878	1.21
Severity of Disorder					
Inpatient Treatment	0.44%	1,550,677	0.80%	189,063	1.83
Outpatient Only	10.00%	1,715,449	12.90%	215,313	1.33
Emergency Room	0.18%	3083	0.22%	473	1.22

Pg: first pregnanacy; PPT: postpartum psychiatric treatment.

**Table 2:** Women with postpartum psychiatric treatments (PPT) relative to first pregnancy outcome (birth or loss) and history of mental health treatments (MHTs) prior to specific pregnancy associated dates.

Time Frame for Identifying Any History of MHT		No Prior Pg Loss		Prior Pg Loss		Crude OR
		*n* = 1,722,250		*n* = 216,828		
		% PPT	Total	% PPT	Total	
One Year	MHT one year prior to first live birth					
	No	7.91%	1,617,780	9.03%	192,511	1.16
	Yes	48.32%	104,470	48.91%	24,317	1.02
	MHT one year prior to first pregnancy outcome					
	No	7.91%	1,617,780	10.42%	209,354	1.35
	Yes	48.32%	104,470	99.97%	7474	>999
	MHT one year prior to first concception					
	No	8.41%	1,630,781	12.47%	209,255	1.55
	Yes	45.06%	91,471	42.00%	7573	0.88
Anytime	MHT any time prior to first conception					
	No	7.07%	1,510,083	8.35%	155,292	1.20
	Yes	33.77%	212,167	26.52%	61,536	0.71
	MHT any time prior to first pregnancy outcome					
	No	7.05%	1,481,689	10.23%	175,053	1.50
	Yes	30.73%	240,561	27.22%	41,775	0.84
	MHT any time prior to first live birth					
	No	7.05%	1,481,689	7.03%	148,891	1.00
	Yes	30.73%	240,561	27.69%	67,937	0.86
Other	MHT between first conception and first live birth					
	No	7.13%	1,614,965	9.38%	196,393	1.35
	Yes	58.99%	107,285	53.09%	20,435	0.79
	Women with MHT prior to first live birth and first MHT					
	Prior to first conception	33.77%	212,167	26.52%	61,536	0.71
	After first conception	8.03%	28,394	38.90%	6401	7.30

**Table 3:** PPT risk regression models for all women (*n* = 1,939,078) controlling for first pregnancy outcome, age, race, and years of live birth, as well as history of mental health treatments (MHT).

Independent Variables Used in Logistic Regression	% PPT	Total	Model 1	Model 2
			Adjuted Odds Ratio (95% Confidence Interval)	Adjusted Odds Ratio (95% Confidence IntervalI)
History of pregnancy loss				
No	10.36%	1,722,250	Ref	Ref
Yes	13.50%	216,828	1.27 (1.25–1.29)	1.10 (1.08–1.12)
Age at live birth (years)				
14–19	12.87%	90,065	Ref	Ref
20–24	9.71%	95,491	0.66 (0.65–0.68)	0.92 (0.90–0.94)
25–29	8.63%	22,098	0.50 (0.50–0.51)	0.82 (0.80–0.83)
Calandar year of live birth				
2000–2002	10.18%	68,717	Ref	Ref
2003–2005	10.83%	54,118	1.01 (1.00–1.03)	0.88 (0.87–0.90)
2006–2008	10.93%	58,032	1.02 (1.00–1.03)	0.82 (0.81–0.83)
2009–2011	11.49%	26,787	1.00 (0.99–1.02)	0.74 (0.72–0.75)
Race				
White	15.86%	123,904	Ref	Ref
Black	9.33%	35,197	0.51 (0.51–0.52)	0.52(0.51–0.52)
Hispanic	5.47%	29,079	0.30 (0.30–0.30)	0.37 (0.37–0.38)
Other	7.83%	19,474	0.45 (0.44–0.46)	0.50 (0.50–0.51)
MHT any time prior to first conception				
No	7.19%	119,683	N/A	Ref
Yes	32.14%	87,971	N/A	5.09 (4.99–5.20)
MHT any time prior to first pregnancy outcome				
No	7.39%	122,353	N/A	Ref
Yes	30.21%	85,301	N/A	1.07 (1.03–1.11)
MHT any time prior to first live birth				
No	7.05%	114,914	N/A	Ref
Yes	30.06%	92,740	N/A	2.13 (2.04–2.22)
MHT occurred between first conception and prior to live birth				
No	7.37%	133,514	N/A	Ref
Yes	58.05%	74,140	N/A	13.39 (13.16–13.62)

MHT = mental health treatment; Ref = reference for comparision in calculating the adjusted odds ratio shown below the reference. Adjusted odds ratio are odds ratios adjusted for the independent variables shown in column one.

**Table 4:** PPT risk regression models for women with a history of pregnancy loss (*n* = 216,828), controlling for age, race, year of live birth (LB), number of losses, type of first loss, pregnancy interval, and history of MHT.

Independent Variables Used in Logistic Regression	% PPT	Total	Model 3	Model 4
			Adj OR (95% CI)	Adj OR (95% CI)
Age at live birth (years)				
14–19	14.24%	96,119	Ref	Ref
20–24	13.13%	97,343	1.11 (1.04–1.18)	1.00 (0.95–1.05)
25–29	12.03%	23,366	1.11 (1.00–1.22)	0.97 (0.92–1.01)
Calendar Year of Live Birth				
2000–2002	13.69%	36,350	Ref	Ref
2003–2005	13.69%	51,142	0.87 (0.83–0.91)	0.83 (0.80–0.87)
2006–2008	13.26%	58,188	0.77 (0.74–0.81)	0.72 (0.69–0.75)
2009–2011	13.47%	71,148	0.69 (0.66–0.72)	0.61 (0.59–0.64)
Race				
White	19.38%	84,731	Ref	Ref
Black	10.60%	62,161	0.54 (0.52–0.56)	0.55 (0.53–0.57)
Hispanic	8.05%	46,334	0.46 (0.44–0.48)	0.45 (0.44–0.47)
Other	10.74%	23,602	0.56 (0.53–0.59)	0.58 (0.55–0.61)
Number of Prior Pregnancy Losses				
1	13.24%	163,993	Ref	Ref
2	14.17%	37,506	1.05 (1.01–1.09)	1.03 (1.00–1.07)
3	14.46%	11,177	1.10 (1.03–1.17)	1.04 (0.98–1.11)
>3	15.20%	4152	1.17 (1.06–1.30)	1.10 (1.00–1.21)
Interval Between First Pg Loss and First LB				
<24 months	13.44%	98,447	Ref	Ref
2–4 years	13.43%	57,669	1.07 (1.03–1.11)	0.97 (0.94–1.01)
4–6 years	13.36%	31,292	1.10 (1.05–1.16)	0.95 (0.91–1.00)
>6 years	14.01%	29,420	1.17 (1.12–1.24)	0.96 (0.91–1.01)
First loss identified as				
Natural loss	13.08%	51,822	Ref	Ref
Induced abortion	13.69%	70,394	0.98 (0.95–1.01)	0.96 (0.93–0.99)
Indeterminate loss	12.70%	41,777	0.98 (0.95–1.02)	0.97 (0.94–1.01)
MHT one year prior to first live birth				
No	9.00%	192,511	Ref	N/A
Yes	48.91%	24,317	1.75 (1.58–1.92)	N/A
MHT one year prior to first conception				
No	12.47%	209,255	Ref	N/A
Yes	42.00%	7573	1.32 (1.25–1.38)	N/A
MHT one year prior to first Pg outcome				
No	10.42%	209,354	Ref	N/A
Yes	99.97%	7474	>999.999	N/A
MHT between first conception and first live birth				
No	12.42%	211,781	Ref	Ref
Yes	59.04%	5047	3.90 (3.53–4.31)	7.25 (6.95–7.56)
MHT any time prior to first live birth				
No	7.03%	148,891	N/A	Ref
Yes	27.69%	67,937	N/A	1.11 (1.03–1.19)
MHT any time prior first conception				
No	8.35%	155,292	N/A	Ref
Yes	26.52%	61,536	N/A	2.29 (2.14–2.45)
MHT any time prior first Pg outcome				
No	10.23%	17,909	N/A	Ref
Yes	27.22%	11,371	N/A	1.09 (1.05–1.14)

## Data Availability

The data used is available from the United States Centers for Medicare and Medicaid Services.
